# Supplementary material for: Content-rich biological network constructed by mining PubMed abstracts
Source: BMC Bioinformatics. 2004 Oct 8;5:147. doi: 10.1186/1471-2105-5-147 (PMC528731; doi:10.1186/1471-2105-5-147)
Supplement: Additional File 2 — The original results of the above study (non-essential files are deleted to keep the file size under the limit set by BMC bioinformatics). [file 1471-2105-5-147-S2.bz2 › chilibotAdditionalFile2/dip05/693ID11994151E18018/html/TOLA_TOLB.html]

 


 **TOLA** and **TOLB** 
  
Found 36 abstracts in PubMed, retrieved 05.  
 

 What does Google say? 
 PDF only 
| .edu only 

---

**Interactive relationship** (e.g. stimulation, inhibition, etc)

**Inhibitory relationship**- The isolation of suppressor mutants of  **tolA**  mutations in the  **tolB**  gene confirmed an interaction between TolAIII and the N terminal domain of  **TolB** .  Ref: 12142433 J Bacteriol, 2002
**Neutral relationship**- Mutational analysis of the  **TolA**  C terminal domain of Escherichia coli and genetic evidence for an interaction between  **TolA**  and  **TolB** .  Ref: 12142433 J Bacteriol, 2002

**Non-interactive relationship** (e.g. studied together, co-existance, homology, etc.)

- Analysis of polar mutations, reverse transcriptase PCR assays, and transcriptional fusion constructs with a promoterless lacZ gene revealed that the genes are arranged in two operons orf1 tolQ tolR  **tolA**   **tolB**  and oprL orf2.  Ref: 12486055 J Bacteriol, 2003
- In Pseudomonas putida, the seven genes, orf1, tolQ, tolR,  **tolA** ,  **tolB** , oprL, and orf2, which encode the proteins of this complex, are clustered in a 5.8 kb region of chromosomal DNA.  Ref: 12486055 J Bacteriol, 2003
- We consider a variety of different colicins, including A, B, D, E1, E3, Ia, M and N, that penetrate through the porins OmpF, FepA, BtuB, Cir and FhuA, to subsequently interact with a few targets in the periplasm, including  **TolA** ,  **TolB** , TolC and TonB.  Ref: 12423783 Biochimie< MedlineTA>Biochimie, 2003
